# Bioinformatics Analysis of miRNAs Targeting TRAF5 in DLBCL Involving in NF-*κ*B Signaling Pathway and Affecting the Apoptosis and Signal Transduction

**DOI:** 10.1155/2022/3222253

**Published:** 2022-12-23

**Authors:** Chunyao Li, Lanshan Huang, Yongqin Wen, Muhua Yi, Min Gao

**Affiliations:** ^1^Department of Pathology, Affiliated Dongguan People's Hospital, Southern Medical University, Dongguan, Guangdong 523000, China; ^2^Departments of Pathology, The First Affiliated Hospital of Guangxi Medical University, Nanning, Guangxi 530021, China; ^3^Patrick G. Johnston Centre for Cancer Research, Queen's University Belfast, Belfast BT9 7AE, UK

## Abstract

**Background:**

Diffuse large B-cell lymphoma (DLBCL) is an aggressive B-cell lymphoma with high heterogeneity. There is an unmet need to investigate valid indicators for the diagnosis and therapy of DLBCL.

**Methods:**

GEO database was utilized to screen for differentially expressed genes (DEGs) and differential miRNAs in DLBCL tissues. The Gene Ontology (GO) and the Kyoto Encyclopedia of Genes and Genomes (KEGG) were applied to analyse DEGs. Then multiple databases were searched for related miRNAs within DLBCL, TNF receptor-associated factor 5 (TRAF5) and NF-kappa B (NF-*κ*B) signaling pathways. The KOBAS database was used to assist in the screening of miRNAs of interest and construct the regulatory network of miRNA-mRNA. Finally, the expression level and diagnostic performance of miRNAs were analyzed with GEO datasets, and DEGs were identified from the GEPIA database.

**Results:**

DEGs were significantly concentrated in the NF-*κ*B signaling pathway and cytokine-cytokine receptor interaction, and involved in the process of immune response and protein binding. MiR-15a-5p, miR-147a, miR-192-5p, miR-197-3p, miR-532-5p, and miR-650 were revealed to be targeting TRAF5 and participating in NF-*κ*B signaling pathway and might impact the apoptosis and signal transduction of DLBCL. In the GEPIA database, TRAF5 was significantly overexpressed in DLBCL. The expression of miR-197-3p was upregulated within GEO datasets, while the rest of the miRNAs were downregulated in DLBCL.

**Conclusions:**

Subsets of miRNAs may participate in the NF-*κ*B signaling pathway by co-targeting TRAF5 and could be prospective biomarkers exploring the pathogenesis of DLBCL.

## 1. Introduction

Diffuse large B-cell lymphoma (DLBCL) is a frequently occurring type of non-Hodgkin lymphoma (NHL) in adults [1]. DLBCL patients have significant heterogeneity in clinical manifestations, pathological characteristics, biological behaviors and molecular genetics, which are a group of highly heterogeneous B-cell malignant lymphoma [1, 2]. Currently, the outcomes of DLBCL patients have been significantly improved due to the contribution of chemotherapy with rituximab combined with cyclophosphamide, doxorubicin, vincristine, and prednisone (R-CHOP) [2, 3]. About 60%–70% of patients might receive long-term remission post first-line treatment, while 30%–40% of patients may experience recurrence or nonresponse, and the median survival time of primary and secondary refractory DLBCL was only 4.4 months [4, 5]. Therefore, the treatment strategies for patients with ineffective first-line treatments, early relapses, or refractory DLBCL have always been challenging in clinic [1]. Exploring the pathogenesis and identifying effective indicators for the diagnosis and treatment of DLBCL are significantly necessary.

In recent years, abnormal stimulation of NF-kappa B (NF-*κ*B) signaling pathway was proposed to be one potential pathogenesis of DLBCL [6, 7]. It has been demonstrated that the activation of NF-*κ*B was caused by mutations in multiple genes, one of which was TNF receptor-associated factor 5 (TRAF5), and the deregulation of TRAF5 may promote the malignant transformation of DLBCL [8]. TRAF5, a signal transduction protein, belongs to the TNF receptor-associated factor (TRAF) family and has been reported to be involved in the positive activation of the NF-*κ*B signaling pathway [9]. However, the function and mechanism of TRAF5 in DLBCL remain unclarified. Aberrant miRNAs were hypothesized to be potential mechanisms initiating the formation and development of B-cell lymphomas, and might even be biomarkers for diagnosis, classification, treatment response or prognosis in primary DLBCL [10]. It is worth noting that TRAF5, miRNAs and their regulatory roles in the NF-*κ*B signaling pathway were rarely reported in DLBCL. With the prevalence of microarray technology and the establishment of various bioinformatics databases, effective tools and novel approaches have been accessible for exploring biomarkers and revealing the mechanisms of tumorigenesis and disease progression [11]. Therefore, this current study aims to explore the relationship of TRAF5, miRNAs, and the NF-*κ*B signaling pathway in DLBCL based on bioinformatics analysis.

In the present research, differentially expressed genes (DEGs) and differential miRNAs of DLBCL were screened out from the GEO database [12], and the DAVID database was utilized to assess the functions and signal pathways of DEGs [13]. Multiple miRNA databases were integrated to search for the DLBCL-related miRNAs, target genes, and signal pathways, and potential miRNA-mRNA regulatory networks were constructed. Finally, GEO datasets were carried out to identify the expression level and diagnostic performance of miRNAs, and DEGs were acquired from the GEPIA database [14]. The present study provided novel lights into the molecular regulation mechanisms of miRNAs and target genes in DLBCL and may facilitate the identification of promising biomarkers for an individualized therapy strategy.

## 2. Materials and Methods

### 2.1. Microarray Data

The GEO database (https://www.ncbi.nlm.nih.gov/geo/) was performed to search for gene and miRNA datasets of DLBCL, with the search term of lymphoma. The inclusion criteria of microarray datasets were as follows: (1) the studies using tissue samples of primary DLBCL from the human species; (2) patients without immune diseases or other tumors, and no relevant treatments received; and (3) nontumor lymphoid tissues as negative controls. The deadline for retrieving the GEO database was June 30, 2021.

### 2.2. Screening of DEGs

In the GEO database, GEO2R (http://www.ncbi.nlm.nih.gov/geo/geo2r) [12] was carried out to analyse the fold change of DEGs in DLBCL patients. In this study, significant DEGs were screened out when |LogFC| (fold change) > 1 and adjusted *P* < 0.05. The DEGs repeated in more than five out of 15 microarrays were selected for subsequent analysis.

### 2.3. GO, KEGG and PPI Analysis of DEGs

In DAVID database (https://david.abcc.ncifcrf.gov/) [13], Gene Ontology (GO) and Kyoto Encyclopedia of Genes and Genomes (KEGG) enrichment analysis were employed to clarify the biological functions of DEGs. Adjusted *P* value < 0.05 was established as the threshold. The STRING database (https://string-db.org/) was utilized to construct protein-protein interaction (PPI) network of DEGs [15].

### 2.4. Retrieval of miRNA Data

(1) The differential miRNAs between the DLBCL group and the control group were analyzed using the GEO2R function in the GEO database. In addition, HMDD (http://www.cuilab.cn/hmdd) [16] and miRWalk (http://mirwalk.umm.uni-heidelberg.de/) [17] were applied to seek for DLBCL-related miRNAs; (2) TRAF5-related miRNAs were acquired from ENCORI (http://www.sysu.edu.cn/) [18], miRWalk, miRDB (http://mirdb.org/) [19] and DIANA-TarBase (http://diana.imis.athena-innovation.gr/DianaTools) [20]; and (3) miRNAs associated with NF-*κ*B signaling pathway were predicted by miRPathDB (https://mpd.bioinf.uni-sb.de/) [21], miRWalk, and DIANA databases. The above miRNA data sets were intersected to obtain hub miRNA sets which concurrently related to the DLBCL, TRAF5, and NF-*κ*B signaling pathway.

### 2.5. Prediction of miRNA Targets and Pathways, and Construction of miRNA-mRNA Network

Targets of hub miRNAs were predicted by 10 databases in five online software program, including ENCORI, miRWalk, miRPathDB, miRDB, and TargetScan (http://www.targetscan.org/) [22]. To reduce the false positive rate, target genes that repeated in more than three out of 10 databases were retained, and miRNAs were selected for further analysis in the case of TRAF5 repeated more than three times. GO and KEGG enrichment of the target genes were analyzed via the KOBAS database (http://kobas.cbi.pku.edu.cn/) [23] (*P* < 0.05). If TRAF5 was concentrated in the NF-*κ*B signaling pathway and involved in apoptosis and signal transduction, the corresponding miRNA was considered to be likely to target TRAF5 and participate in the NF-*κ*B signaling pathway. And then the corresponding miRNA and its target genes involved in NF-*κ*B signaling pathway were integrated. Finally, the miRNA-mRNA regulatory network involved in NF-*κ*B signaling pathway was obtained, and Cytoscape was used for visualization [24].

### 2.6. Expression of TRAF5 and miRNAs

The relative expression quantity of miRNAs in the GEO database was extracted. Differential expression levels of miRNAs were identified between the DLBCL group and the control group, and receiver operating characteristic (ROC) curve was utilized to evaluate diagnostic performance of miRNAs. Meanwhile, differential expression of TRAF5 and other target genes between DLBCL and the control group was identified using the GEPIA database (http://gepia.cancer-pku.cn/) [14].

### 2.7. Statistical Analysis

SPSS 22.0 was applied for data analysis. Graphpad Prism 7.0 was used for plotting. Statistical significance of each two groups was calculated by the Student's *t*-test. *P* < 0.05 revealed statistical significance.

## 3. Results

### 3.1. Screening Results of DEGs

**Figure 1:**
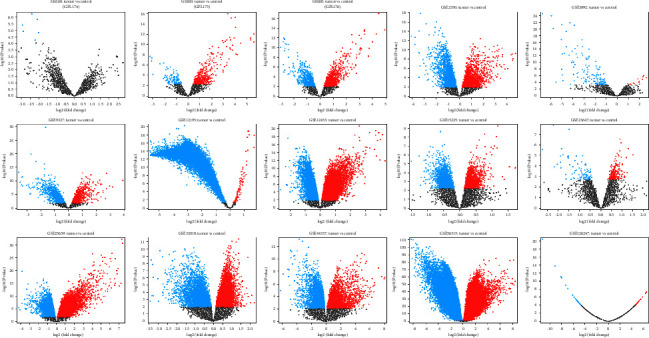
Volcano plots of DEGs detected in 15 datasets from GEO database. Red, blue, and black dots denote upregulated genes, downregulated genes, and nonsignificant genes, respectively.

A total of 15 gene expression profile datasets were screened out from GEO database (GSE60-GPL174 [25], GSE60-GPL175 [25], GSE60-GPL176 [25], GSE2350-GPL91 [26], GSE3892 [27], GSE9327 [28], GSE12195 [8], GSE12453 [29], GSE15225 [30], GSE23647 [31], GSE25639 [32], GSE32018 [33], GSE44337 [34], GSE56315 [35], and GSE126247 [36]) (Figure 1), including 20034 DEGs. In order to reduce the false positive rate, a total of 243 DEGs that appeared in more than five microarrays were selected for subsequent analysis.

### 3.2. Enrichment Analysis of DEGs

**Figure 2:**
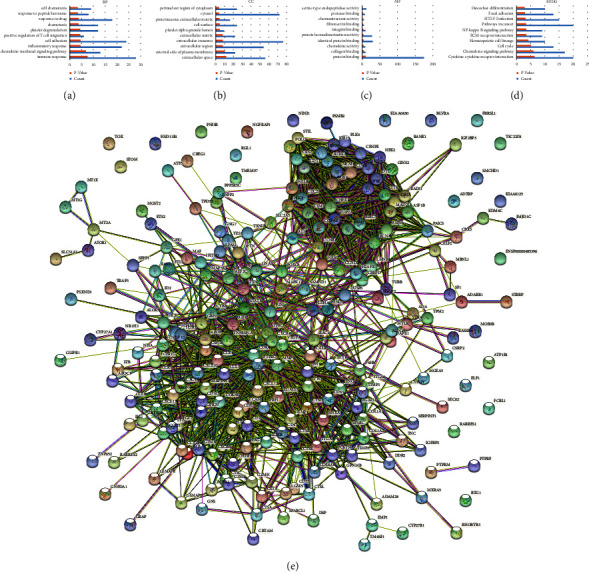
GO, KEGG, and PPI analyses of 243 DEGs. (a-c) GO functional enrichment analysis (top 10), including BP, CC, and MF. (d) KEGG pathway enrichment analysis (top 10). (e) PPI network of DEGs.

The biological process (BP) of the 243 DEGs was mainly focused on the chemokine-mediated signaling pathway, immune response, cell adhesion and inflammatory response. The cellular component (CC) was mainly existed in the extracellular region, external side of plasma membrane, cytosol, and cell surface. Molecular function (MF) was primarily involved in protein binding, chemokine activity, and protein homodimerization activity (Figures 2(a)–2(c)). Furthermore, KEGG pathway was markedly enriched in hematopoietic cell lineage, cytokine-cytokine receptor interaction, ECM-receptor interaction, chemokine signaling pathway, and the NF-*κ*B signaling pathway (Figure 2(d)). The PPI network of DEGs-encoded proteins was displayed in Figure 2(e). In addition, TRAF5 was identified to be participated in the NF-*κ*B signaling pathway and being involved in apoptosis and signal transduction in BP. The CC of TRAF5 was distributed in the cytosol, and the MF of TRAF5 was associated with protein binding.

### 3.3. Screening Results of miRNAs

**Figure 3:**
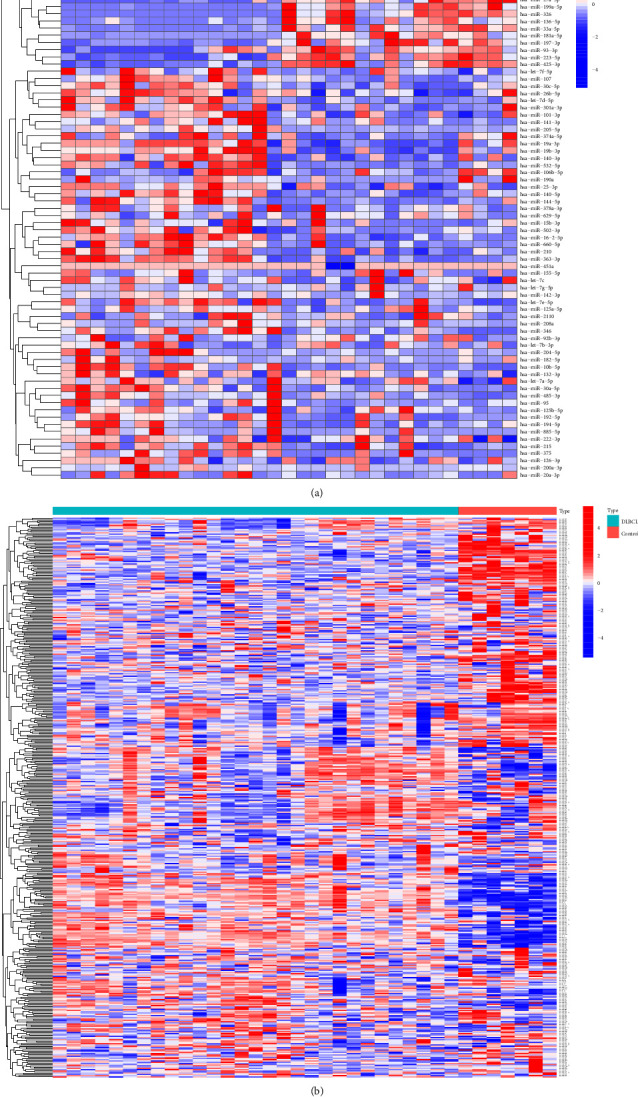
Heatmaps of significantly expressed miRNAs in GEO datasets. (a) 76 miRNA expression profiles in GSE117063. (b) 457 miRNA expression profiles in GSE29493.

**Figure 4:**
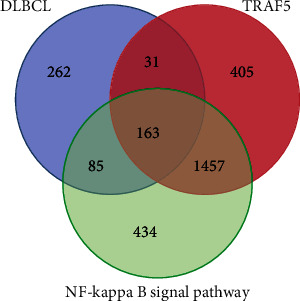
Intersection of miRNAs associated with DLBCL, TRAF5, and NF-kappa B signaling pathway.

(1) A total of 541 differentially DLBCL-related miRNAs were obtained from GEO datasets (GSE29493 and GSE117063), HMDD, and miRWalk databases. All miRNAs with statistical significance from GEO datasets were shown in Figure 3; (2) There were 2056 miRNAs associated with TRAF5 that were predicted in ENCORI, miRWalk, DIANA TarBase, and miRDB databases; (3) A total of 2139 miRNAs connected with NF-*κ*B signaling pathway were selected from the DIANA, miRWalk, and miRPathDB databases. After the intersection of the above three miRNA data sets, 163 miRNAs that were concurrently related to the DLBCL, TRAF5, and NF-*κ*B signaling pathway were obtained (the Venn diagram is shown in Figure 4).

### 3.4. Prediction and Enrichment Analysis of miRNA Target Genes

**Figure 5:**
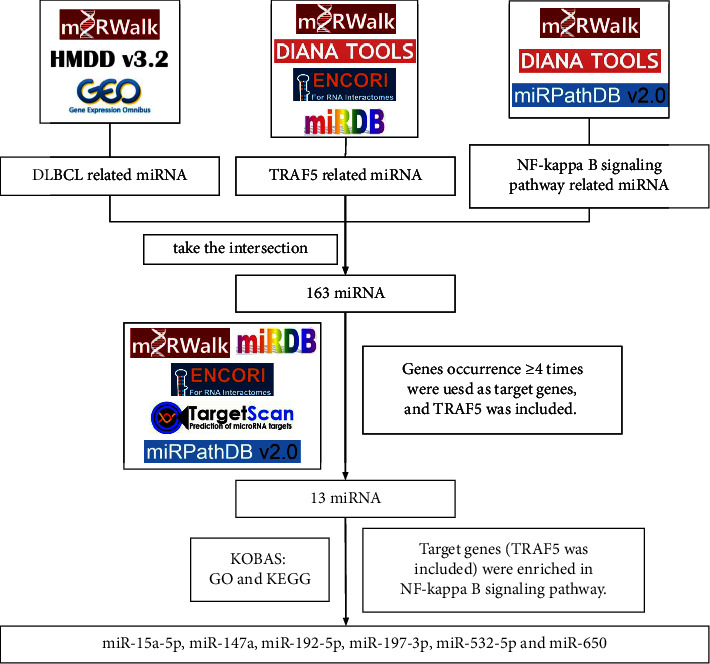
Flow chart of miRNAs screening that target TRAF5 in DLBCL and participate in NF-kappa B signaling pathway.

**Figure 6:**
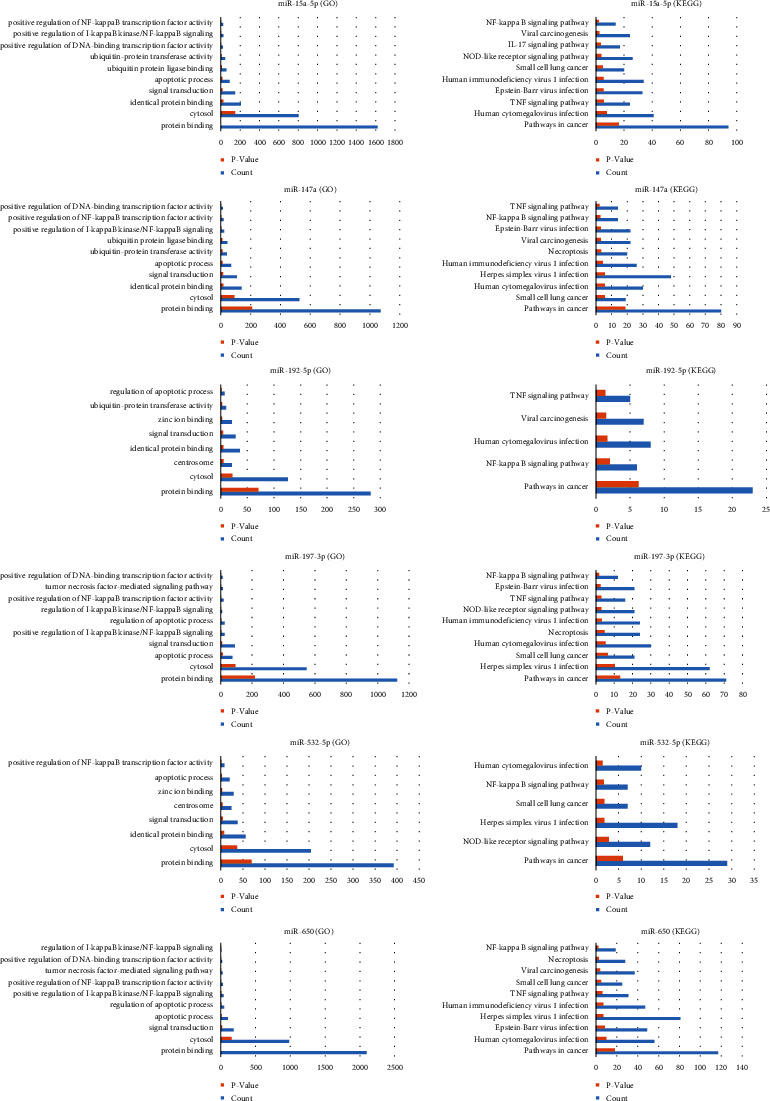
GO and KEGG enrichment analyses of TRAF5 in miR-15a-5p, miR-147a, miR-192-5p, miR-197-3p, miR-532-5p, and miR-650 using KOBAS.

Target genes of 163 miRNAs were predicted using ENCORI, miRWalk, miRPathDB, TargetScan, and miRDB. The miRNAs were selected for further analysis in the case of TRAF5 repeated more than three times, and the target genes were retained, which were predicted by more than three databases. In the KOBAS database, the target genes (including TRAF5) of miR-15a-5p, miR-147a, miR-192-5p, miR-197-3p, miR-532-5p, and miR-650 were all simultaneously enriched in the NF-*κ*B signaling pathway (the screening process is provided in Figure 5). In the GO analysis, TRAF5 was involved in apoptosis and signal transduction in all six miRNAs. The function and pathway analysis of TRAF5 in miRNAs are summarized in Figure 6.

### 3.5. Analysis of the miRNA-mRNA Network

**Figure 7:**
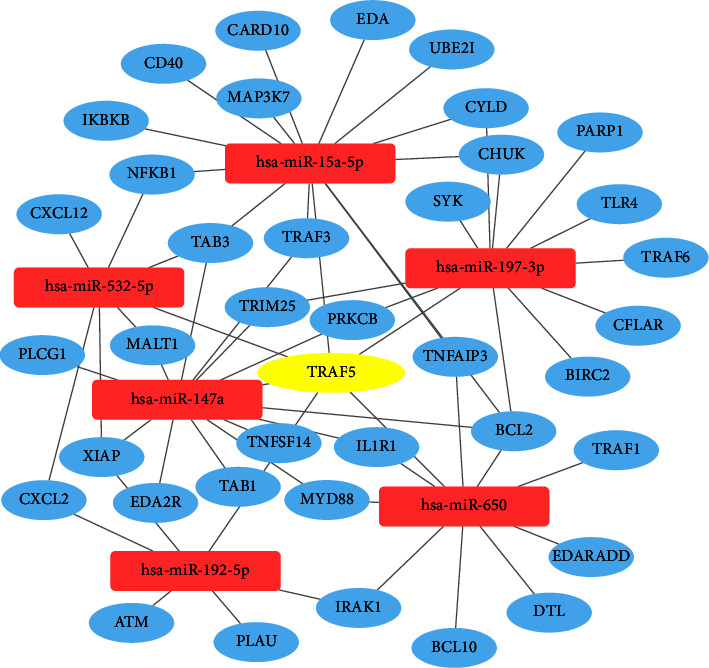
The miRNA-mRNA regulatory network of miR-15a-5p, miR-147a, miR-192-5p, miR-197-3p, miR-532-5p, miR-650, and the targeted genes participating in NF-kappa B signaling pathway. Rectangle nodes represent miRNAs, and oval nodes represent mRNAs.

MiR-15a-5p, miR-147a, miR-192-5p, miR-197-3p, miR-532-5p, and miR-650 with corresponding target genes involved in the NF-*κ*B signaling pathway were constructed into a visualized miRNA-mRNA regulatory network (Figure 7). In addition to targeting TRAF5, miRNAs also participated in the NF-*κ*B signaling pathway by regulating other 38 target genes, such as myeloid differentiation primary response 88 (MYD88), X-linked inhibitor of apoptosis (XIAP), B-cell lymphoma 2 (BCL2), and interleukin-1 receptor-associated kinase 1 (IRAK1).

### 3.6. Expression of miRNA and TRAF5 in DLBCL

**Figure 8:**
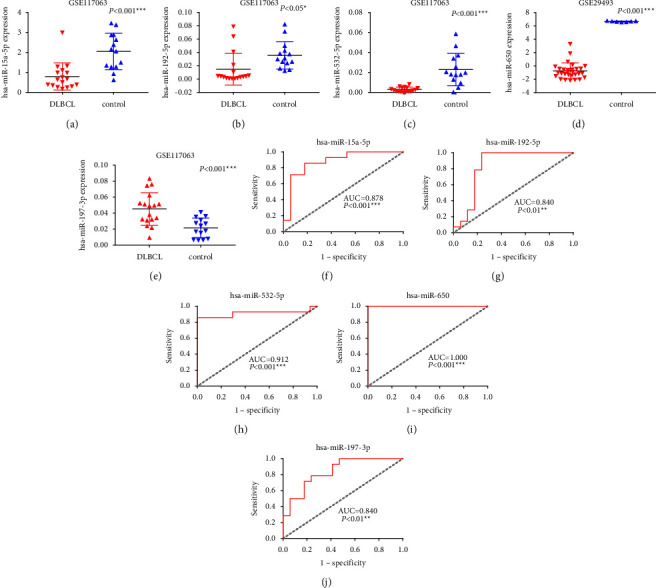
Scatter diagrams and ROC curves analysis for the differential expression of miRNAs between the DLBCL and control group in GEO database. (a–d) MiR-15a-5p, miR-192-5p, miR-532-5p, and miR-650 were downregulated in DLBCL. (e) MiR-197-3p was upregulated in DLBCL. (f–j) ROC curves presented excellent diagnostic performance of each miRNA. ^∗^*P* < 0.05, ^∗∗^*P* < 0.01, ^∗∗∗^*P* < 0.001.

**Figure 9:**
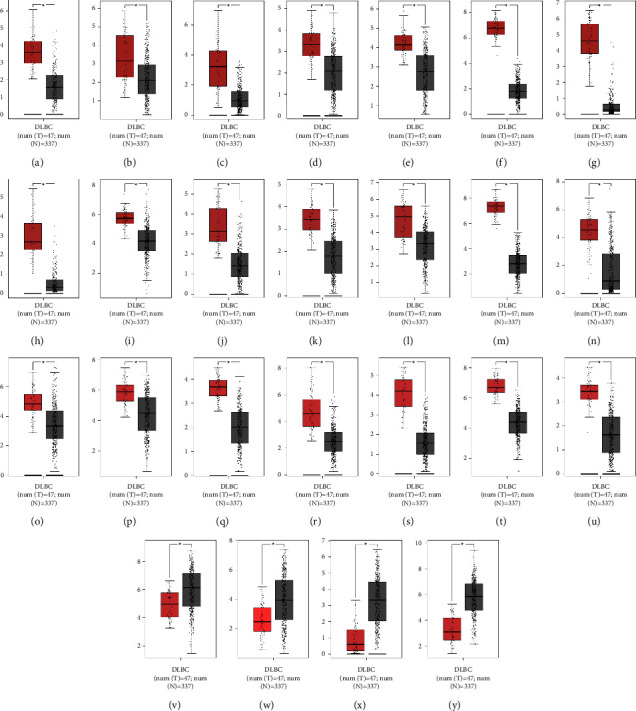
GEPIA database analyzed mRNA expression levels associated with target genes that participated in NF-kappa B signaling pathway. (a–u) TRAF5, ATM, BCL2, BCL10, BIRC2, CD40, CXCL12, DTL, IRAK1, MALT1, MAP3K7, NFKB1, PARP1, PLAU, PLCG1, SYK, TAB1, TRAF1, TRAF3, UBE2I, and XIAP were significantly increased in DLBCL. (v–y) MYD88, TLR4, TNFSF14 and TRIM25, notably, decreased in DLBCL. The red color represents DLBCL samples, and the gray color represents normal control samples. ^∗^*P* < 0.05.

Information of screened miRNAs of the two GEO datasets are displayed in Table 1. In GEO database, miR-15a-5p, miR-192-5p, miR-532-5p, and miR-650 were downregulated in DLBCL tissues (Figures 8(a)–8(c)), while miR-197-3p was highly expressed in DLBCL compared with the control group (Figure 8(d)). ROC curves indicated that the above miRNAs displayed high diagnostic value in DLBCL, and the area under curve (AUC) were 0.878 (*P* < 0.001), 0.840 (*P* < 0.01), 0.912 (*P* < 0.001), 1.000 (*P* < 0.001), and 0.840 (*P* < 0.01), respectively (Figures 8(e)–8(h)). In GEPIA database, higher expression of TRAF5 was identified in DLBCL than in the control group (Figure 9(a)). Meanwhile, other target genes of miRNAs participating in NF-*κ*B signaling pathway were also analyzed, and a total of 20 target genes were overexpressed in DLBCL, such as BCL2, IRAK1, and XIAP (Figures 9(b)–9(u)). In contrast, four target genes were presented with inverse expression in DLBCL, including MYD88, toll like receptor 4 (TLR4), TNF superfamily member 14 (TNFSF14) and the tripartite motif containing 25 (TRIM25) (Figures 9(v)–9(y)).

## 4. Discussion

In recent years, variations, trends, and associations of tumor samples can be rapidly accessed through computational investigations and bioinformatics, which have enhanced the discovery and analysis of the mutual regulation of miRNAs, target genes, and signaling pathways in tumors [11]. In the research of malignant mesothelioma (MM) [37], miR-323a-3p was upregulated in mesothelial cell models of pleura which exposed to fluoro-edenite fibers, and miR-101-3p was downregulated in MM cell, which were in concordance with GEO datasets. In subsequent studies of tissue samples, the translational data of miR-101-3p was consistent with previous bioinformatics analysis, which revealed significantly low expression in MM [38]. Chen et al. [39] found eight key miRNAs and 14 genes of colon cancer via bioinformatics analysis, and most of which have been verified in previous experiments. In addition, downregulated miRNAs and overexpressed genes were screened from the online analysis of Hepatitis B virus-related hepatocellular carcinoma, and the results were consistent with the real-time PCR and previous laboratory findings [40]. Overall, these studies have provided evidence of the reliability and feasibility of bioinformatics analysis for clinical tumors.

The molecular mechanisms of DLBCL have been intensively studied, but the pathogenesis of DLBCL is intricacy, and the relationships among molecular markers, signaling pathways, and regulatory mechanisms are complicated [1, 2]. Bioinformatics has been widely applied to data mining, which revealed great significance in exploring the pathogenesis and precise treatment strategies of DLBCL [41]. On the basis of GEO datasets, B-cell lymphoma 6 (BCL6) was identified as a target gene of miR-30 and involved in ibrutinib resistance of DLBCL, and cell experiment was consistent with the computational result [42]. In the study by Li et al. [43], overexpression of cyclin D2 (CCND2) and activation of the Wnt pathway in the activated B-cell (ABC) subtype of DLBCL were validated by bioinformatics and experiments. Our study revealed that TRAF5 was involved in apoptosis, signal transduction, and protein binding of DLBCL. TRAF5 participated in the NF-*κ*B signaling pathway together with C-X-C motif chemokine ligand 12 (CXCL12), C-C motif chemokine ligand 21 (CCL21), interleukin 1 receptor type 1 (IL1R1), vascular cell adhesion molecule 1 (VCAM1), CCL4, ATM serine/threonine kinase (ATM), lymphotoxin beta (LTB), and TNF receptor superfamily member 1A (TNFRSF1A).

The NF-*κ*B signaling pathway is one of the crucial regulatory mechanisms of apoptosis and participates in multiple stages of B lymphocytes development [6]. In lymphoma, the continuous excitation of NF-*κ*B can prevent cell differentiation, inhibit apoptosis, and promote proliferation of tumor cells, and meanwhile increase inflammatory response, tumor microvascular formation, and metastasis [44–46]. More recently, a number of studies revealed that micromolecule targeted inhibitors could suppress tumor progression via restraining NF-*κ*B signaling [47–49]. Therefore, it is thought to be a promising therapeutic target to block the abnormal stimulation of the NF-*κ*B signaling pathway in cancer. The abnormalities of MYD88, mucosa associated lymphoid tissue lymphoma translocation gene 1 (MALT1), B-cell lymphoma 10 (BCL10), and other functional driver genes may lead to the excitation of the NF-*κ*B signaling pathway in DLBCL, promoting proliferation of tumor cells, and ultimately leading to tumorgenesis of DLBCL [50]. In Compagno group's study [8], the activation of NF-*κ*B in DLBCL was aroused by multiple genes, including TRAF5, NF-*κ*B inhibitor alpha (NFKBIA) and TNF receptor-associated factor 3 (TRAF3). To a certain extent, this experimental research verified the reliability of our calculation results. The missing or activated genes may accelerate the occurrence of lymphoma via causing wrong stimulation of NF-*κ*B signaling pathway [6]. However, the functional consequences of these disordered genes in DLBCL have not been thoroughly identified.

Previous studies have demonstrated that TRAF5 could bind to cell membrane or intracellular receptors such as CD30, CD40, and latent membrane protein 1 (LMP1) and stimulate downstream related signal transduction pathways [51]. The stimulation ultimately activates NF-*κ*B signaling pathway and performs a variety of significant functions in regulating cell cycle, inhibiting apoptosis, participating in inflammatory response, and immune regulation [9, 51, 52]. However, TRAF5 has rarely been reported in lymphoma. Guo et al. [53] found that TRAF5 mRNA was more highly expressed in Hodgkin's lymphoma (HL) cell line than that in normal B lymphocytes, and the aberrant expression of CD30 inhibited the expression of endogenous TRAF5 protein. Horie et al. [54] proved that TRAF5 assembled in the cytoplasm of H-RS cells in HL and also congregated near the cell membrane together with CD30, indicating that TRAF5 and CD30 were jointly mediated the activation of NF-*κ*B. The study of Sutherland et al. [55] has clarified that TRAF5 could combine with the cytoplasmic domain of CD40 to activate NF-*κ*B signaling pathway, and then inhibit apoptosis in B lymphoma cells of mouse. The above studies indicated that TRAF5 was aberrantly overexpressed in lymphoma. In our current study with GEPIA data, the overexpression of TRAF5 was discovered in DLBCL tissues, indicating that TRAF5 may be a promising molecular target and play potential roles in DLBCL. However, further research is warranted to look into the mechanism of TRAF5 in DLBCL.

As we know, the abnormal expression of miRNAs can change the production of target mRNAs and the expression of downstream proteins, leading to defects in the cell cycle and apoptosis mechanisms [56]. In this current study originating from GEO data, the expression of miR-15a-5p, miR-192-5p, miR-532-5p, and miR-650 were extremely lower in DLBCL compared with the control group, while miR-197-3p was of high expression in DLBCL, and all miRNAs showed dominant diagnostic value. These results might require further validation by clinical patient cohorts, and detection of the expression level of miRNAs through body fluids could be one of the prospective approaches in the era of liquid biopsy. Based on bioinformatics analysis, these miRNAs were identified to participate in the NF-*κ*B signaling pathway by co-targeting TRAF5, thereby affecting apoptosis and signal transduction of DLBCL. However, the mechanism of each miRNA has not been thoroughly investigated in DLBCL. In the study of nucleus pulposus cells of mouse, Zhang et al. [57] found the downregulation of miR-15a-5p inhibited inflammation and apoptosis through NF-*κ*B pathway. Overexpression of miR-147a exhibited poor prognosis in hepatitis C virus-positive DLBCL patients [58]. Li and Huang [59] demonstrated that upregulation of miR-147a inhibited the MyD88/TRAF6/NF-*κ*B pathway to alleviate endothelial cell damage induced by high glucose. The higher expression of miR-532-5p was reported in previous research on the plasma of DLBCL patients [60]. In addition, Zhang et al. [61] clarified that decreased miR-532-5p expression indicated a poor prognosis of gastric cancer, and the inhibition of miR-532-5p promoted angiogenesis, metastasis, and NF-*κ*B activity. MiR-650 was also found to be upregulated in glioma, inducing the activation of NF-*κ*B pathway, and promoting migration, proliferation, and invasion of glioma cells [62]. Hence, miRNAs play vital roles in various diseases, and its abnormal expression in DLBCL may activate NF-*κ*B signaling pathway via stimulating the activation of multiple target genes. At last, miRNAs also presented to target other 38 genes, such as BCL2, XIAP, IRAK1, and MYD88, to participate in the NF-*κ*B signaling pathway based on our analysis of the miRNA-mRNA network. This complex regulatory network may participate in the malignant transformation of B lymphocytes and ultimately lead to the occurrence and development of DLBCL.

There are several limitations for current study. First, the GEO2R online tool of the GEO database was utilized for data analysis in order to minimize the heterogeneity of results during the integration of differentially expressed genes. Although GEO is the most comprehensive and commonly used database, which data is always shared with other databases, there are still some datasets not included due to distinct experimental platforms, designs, materials, sequencing methods, and data types. Second, the current bioinformatics analysis was conducted as a preliminary study without validation by clinical cohorts or experimental research. Clinical, in vivo, and in vitro research are needed to further support our findings.

## 5. Conclusion

In conclusion, this study indicated that miR-15a-5p, miR-147a, miR-192-5p, miR-197-3p, miR-532-5p, and miR-650 might participate in NF-*κ*B signaling pathway by co-targeting TRAF5 and affect the apoptosis and signal transduction of DLBCL. This study provides a theoretical basis for in-depth research on the molecular regulation mechanism of DLBCL and provides evidence to explore new diagnostic biomarkers and cancer-targeted therapeutic drugs. However, these results still need to be verified by extensive experiments.

## Figures and Tables

**Table 1:** Information of screened miRNAs in GEO datasets.

miRNA ID	logFC	*P* value	Adj *P*val	Platform	GEO ID	
hsa-miR-15a-5p	−1.59	5.29 *E* − 05	3.40 *E* − 04	GPL25327	GSE117063	Downregulated
hsa-miR-192-5p	−2.5126	1.20 *E* − 04	6.72 *E* − 04	GPL25327	GSE117064	Downregulated
hsa-miR-197-3p	1.1644	1.11 *E* − 03	3.57 *E* − 03	GPL25327	GSE117065	Upregulated
hsa-miR-532-5p	−2.9023	2.68 *E* − 04	1.29 *E* − 03	GPL25327	GSE117066	Downregulated
hsa-miR-650	−7.4085	9.56 *E* − 21	1.49 *E* − 20	GPL9081	GSE29493	Downregulated

## Data Availability

The datasets used and/or analyzed in this study are available from the corresponding author upon reasonable request. Partial data used and analyzed during this study are available from the GEO (https://www.ncbi.nlm.nih.gov/geo/) and GEPIA (http://gepia.cancer-pku.cn/).
